# Persistent Foot-and-Mouth Disease Virus Infection in the Nasopharynx of Cattle; Tissue-Specific Distribution and Local Cytokine Expression

**DOI:** 10.1371/journal.pone.0125698

**Published:** 2015-05-21

**Authors:** Juan M. Pacheco, George R. Smoliga, Vivian O’Donnell, Barbara P. Brito, Carolina Stenfeldt, Luis L. Rodriguez, Jonathan Arzt

**Affiliations:** 1 Plum Island Animal Disease Center, Foreign Animal Disease Research Unit, Agricultural Research Service, United States Department of Agriculture, Plum Island, NY, United States of America; 2 Department of Pathobiology and Veterinary Science, University of Connecticut at Storrs, Storrs, CT, United States of America; 3 Center for Animal Diseases Modeling and Surveillance, University of California Davis, Davis, CA, United States of America; Friedrich-Loeffler-Institut, GERMANY

## Abstract

Tissues obtained post-mortem from cattle persistently infected with foot-and-mouth disease virus (FMDV) were analyzed to characterize the tissue-specific localization of FMDV and partial transcriptome profiles for selected immunoregulatory cytokines. Analysis of 28 distinct anatomic sites from 21 steers infected with FMDV serotype A, O or SAT2, had the highest prevalence of overall viral detection in the dorsal nasopharynx (80.95%) and dorsal soft palate (71.43%). FMDV was less frequently detected in laryngeal mucosal tissues, oropharyngeal mucosal sites, and lymph nodes draining the pharynx. Immunomicroscopy indicated that within persistently infected mucosal tissues, FMDV antigens were rarely detectable within few epithelial cells in regions of mucosa-associated lymphoid tissue (MALT). Transcriptome analysis of persistently infected pharyngeal tissues by qRT-PCR for 14 cytokine genes indicated a general trend of decreased mRNA levels compared to uninfected control animals. Although, statistically significant differences were not observed, greatest suppression of relative expression (RE) was identified for IP-10 (RE = 0.198), IFN-β (RE = 0.269), IL-12 (RE = 0.275), and IL-2 (RE = 0.312). Increased relative expression was detected for IL-6 (RE = 2.065). Overall, this data demonstrates that during the FMDV carrier state in cattle, viral persistence is associated with epithelial cells of the nasopharynx in the upper respiratory tract and decreased levels of mRNA for several immunoregulatory cytokines in the infected tissues.

## Introduction

Foot-and-mouth disease (FMD) is caused by FMD virus (FMDV), a member of the *Aphthovirus* genus in the *Picornaviridae* family [1]. FMD is one of the major constraints to international trade in animal products due to its extreme contagiousness and broad spectrum of host species that includes wild and domesticated ruminants and suids. Control and regional eradication of FMDV is complicated by the existence of seven serotypes and several subtypes, transmissibility by air, and occurrence of a prolonged asymptomatic carrier state in a large proportion of infected ruminants. Although the role of persistently infected ruminants in disease transmission remains unclear, the international standards on FMD from the World Organization for Animal Health (OIE) indicate that in order to regain FMD-free status countries must demonstrate freedom of FMD virus infection [2]. Thus asymptomatic FMDV carrier animals are perceived as a threat and the existence of the carrier state complicates regaining FMD-free status. On this basis, FMD-free countries generally will maintain trade barriers for animals and animal products from countries that have not demonstrated absence of FMDV including absence of carrier animals. [3–5]

VanBekkum et al [6] were first to document the presence of infectious FMDV in oropharyngeal fluid (OPF) of asymptomatic cattle several weeks after infection. This was later confirmed by Sutmoller and Gaggero [7]. FMDV carrier animals were subsequently defined as ‘any animal from which FMDV can be recovered in oropharyngeal scrapings using a probang sampling cup during periods greater than 28 days post infection (dpi)’ [8]. FMDV persistence has been demonstrated to occur in cattle, sheep, goats, Asian buffalo, and various wildlife species [9] most notably the African buffalo [10]. Persistence occurs with variable incidence regardless of FMDV vaccination status, clinical outcome of infection, challenge strain and dose, and host factors including sex and age (reviewed by Salt [5]). Various mechanisms have been proposed to explain the inability of some animals to clear the virus including variations in the kinetics of the host response to FMDV infection [11], viral mutation leading to antigenic variation [12], or differences in the innate immune response [13]. However, the mechanism(s) responsible for the establishment, maintenance, and resolution of the carrier state remain undetermined.

Various published works have implicated the pharyngeal tissues of cattle as the predilection site for FMDV persistence [13–17]. Similarly, several investigators have confirmed that collection of oropharyngeal sputum with a probang device was an effective manner of recovery of FMDV from carriers [6,7,14,18]. However, this technique is incapable of indicating the identity of the specific tissues or cells within which the virus persists. Burrows was first to perform tissue-specific isolation of virus on FMDV carriers to identify the dorsal soft palate (i.e. floor of nasopharynx) as the site with greatest frequency of virus recovery and highest mean infectivity [14]. Furthermore, cell cultures derived from the pharynx of persistently infected cattle have been shown to remain FMDV-positive [19,20]. Persistence can also be established in pharyngeal primary cell cultures from uninfected animals by FMDV infection *ex vivo* [21].

Microscopic localization of FMDV RNA in the basal layers of the epithelium of the dorsal soft palate (DSP) and pharynx has been demonstrated by in situ hybridization [16,17]. Additionally, FMDV RNA and antigens have been detected in lymphoid germinal centers in pharyngeal MALT tissues and lymph nodes of cattle at 38 days after challenge without concurrent detection in pharyngeal epithelia [15]. In a more recent study, Stenfeldt et al described the detection of low levels of FMDV RNA in biopsy samples of pharyngeal epithelia during persistent phases of infection. The lower FMDV RNA content compared to probang samples led to the conclusion that the targeted biopsy area within the DSP does not consistently harbor FMDV replication during persistent infection [22].

It is well documented that FMDV subverts the early immune response particularly by targeting innate immune mechanisms (reviewed by Golde *et al* [23]). This occurs through inhibitory effects on cytokine-driven pathways resulting in impaired function of antigen presenting cells and their precursors (reviewed by Grubman *et al* [24]). This ultimately could lead to suboptimal immune function favoring viral replication and delaying onset of specific adaptive T-cell response. Interferon (IFN) types I, II, and III have been demonstrated to impair FMDV replication *in vitro* and *in vivo* [25]. In order to successfully replicate, FMDV blocks expression of IFN and other IFN-stimulated genes (ISGs) [25]. However, *in vivo* replication will induce synthesis of substantial levels of INF I/III [26–29]. Other cytokines including IP-10, IL-2, IL-4, IL-10, IL-12, IL-15 and IL-18 have been shown to affect FMDV replication through dendritic cells (DC) activation and maturation and NK cell recruitment, proliferation and activation (as reviewed by Toka *et al* [30]). Additionally, IL-1, IL-6, TNF-α, IL-10 [31] and RANTES [32] have also been associated with playing a role in controlling FMDV replication and spread.

There have been few published descriptions regarding cytokines involved in the host response during the carrier stage. Relative expression levels of IFN-α and -β below baseline, but without statistical significance, have been described in persistently infected cattle [13]. It has also, been described that IFN-γ might have an effect in the development of persistent infection *in vitro* [20,21] and *in vivo* [33]. Additionally, increased levels of TNF-α mRNA have been described in association with FMDV persistence in cattle [13,34]. However, none of these mechanisms have been demonstrated to represent a defining event in establishment of persistent infection.

The current study investigated tissue-specific localization of FMDV in persistently infected cattle to provide a detailed anatomic map of the distribution of FMDV in persistently infected tissues. In addition transcriptome profiles for selected immunoregulatory cytokines are reported for these same tissues to suggest potential mechanisms of viral persistence. The overall conclusion was that the nasopharyngeal mucosal tissues were the most frequent sites of FMDV persistence and a trend of suppression of cytokine mRNA expression was identified.

## Materials and Methods

### Experimental animals, virus, and inoculation systems

All experimental procedures were subjected to prior approval by Plum Island Animal Disease Center's Institutional Animal Care and Use Committee, which functions to ensure ethical and humane treatment of experimental animals. This included daily monitoring of the health of the experimental animals. Whenever excessive pain was observed, steps were taken to minimize animal suffering by delivery of analgesics and/or anti-inflammatory drugs at 1.1–2.2 mg per kg of flunixin meglumine every 12–24 h and/or 0.1 mg/kg of butorphanol tartrate every 8–24 h. If pain could not be pharmacologically controlled, animals were humanely euthanized. For virus inoculation, steers were sedated using Xylazine, IM, 0.22 mg/kg. Sedation was reversed with Tolazoline, IV, 2 mg/kg. Euthanasia was performed after sedation with Xylazine utilizing Fatal-plus, IV, 10 mL/45.3 kg. All experimental subjects were 9 to 12 month-old Holstein steers weighing 400–500 kg that were obtained from an AAALAC (Association for Assessment and Accreditation of Laboratory Animal Care International)-accredited experimental-livestock provider (Thomas-Morris Inc., Reisterstown, MD). For all experiments, animals were housed in a BSL-3Ag animal facility from time of inoculation or vaccination until time of euthanasia. Steers were recruited for the current study after primarily serving in pathogenesis or vaccination-challenge studies which are not described herein.

Animals were infected with FMDV strains of serotypes A, O and SAT2 with or without previous homologous vaccination. In all groups, challenge was performed by intradermolingual or contact inoculation as previously described [35]. In order to identify FMDV carriers, 46 convalescent steers were screened beyond 27 dpi by probang sampling followed by FMDV rRT-PCR and virus isolation (VI). An FMDV carrier was defined as a steer from which infectious FMDV was recovered from oropharyngeal fluid or tissue at ≥28 dpi. Infectious FMDV is defined as virus that is able to replicate in cell culture, with or without pre-treatment of the sample with triclorotrifluoroethane (TTE) (as described below for probang processing). On the basis of this definition, 21 carrier steers were selected for further investigation as described below (Table 1). Experiments were terminated at predetermined end points at 28 to 52 dpi.

**Table 1:** Antemortem results of 21 FMDV persistently infected cattle.

Strain	A24 RGD							A24 SGD							O1 Manisa ^a^			SAT2			
Steer #	392	393	395	242	400	020	244	233	755	560	207	199	004	030	626	625	624	612	613	614	618
Route of inoculation	IDL ^b^	IDL	CT ^c^	IDL	IDL	CT	CT	IDL	IDL	IDL	CT	CT	IDL	IDL	CT	IDL	IDL	IDL	IDL	IDL	IDL
Necropsy at DPI ^d^	41	41	41	35	35	36	36	28	28	29	44	44	42	42	37	37	40	44	44	45	52
FMDV Vaccinated	No	No	No	No	No	No	No	No	No	No	No	Yes	Yes	Yes	No	No	No	Yes	Yes	Yes	Yes
Dose of inoculum (log_10_ BTID_50_)	4.0	4.0	NA ^e^	4.0	4.0	NA	NA	3.1	1.1	1.7	NA	NA	4.0	4.0	NA	4.0	4.0	4.0	4.0	4.0	4.0
First clinical signs ^f^ (DPD ^g^/DPC ^h^)	2	6	4	2	ND ^i^	3	3	2	2	2	≤4	ND	ND	≤4	2	1	1	ND	ND	ND	≤6
Viremia range (DPD/DPC)	1–3	1–3	3–5	1–3	1–3	2–5	4–5	NE ^j^	NE	NE	4	ND	ND	ND	3–5	1–3	1–3	ND	ND	ND	2
Results of probang testing ^k^	29,PP	29,PP	29,PP	28,PP	28,PP	28,PP	28,PP	27,PP	27,PP	27,PP	28,PP	28,PP	28,NN	28,PP	34,PP	34,PP	34,PP	28,PP	28,PP	28,PP	28,PP
	33,PP	33,PP	33,PP	29,PP	29,PP	29,NP	29,NP			28,PP	35,PP	35,NP	31,PP	31,PP	35,PP	35,PP	35,PP	29,PN	29,PP	29,PP	29,NP
	34,PP	34,PP	34,PP	30,NP	30,PP	30,NP	30,NP			29,PP	42,NP	42,PP	35,NP	35,PP	36,NP	36,NN	36,PP	30,PP	30,PP	30,PN	30,NP
	35,PP	35,PP	35,PP	31,NP	31,PP	31,PP	31,NP						38,PP	38,PP			37,PP	31,PP	31,PP	31,PP	31,NP
	41,PP	41,PP	41,PP	34,PP	35,PP	35,PP	34,PP										40,PP	32,PP	32,PP	32,PN	32,NP
				35,PP		36,NP	35,PP											39,PN	39,PP	39,PP	39,PP
							36,NP											42,PP	42,PP	42,PP	42,PP
																		43,PP	43,PP	43,PN	43,PP
																			44,PP	44,PP	44,PP
																				45,NN	45,PP
																					46,PN

^a^ Previously described in Pacheco, Arzt, and Rodriguez, 2010

^b^ IDL: intradermolingual (direct inoculated)

^c^ CT: contact inoculated

^d^ DPI: day post inoculation

^e^ NA: Not applicable

^f^ Starting day of lesions in other than inoculation site

^g^ DPD: Day post direct inoculation

^h^ DPC: Day post contact inoculation

^i^ ND: Not detected

^j^ NE: Not evaluated

^k^ Number indicates day post inoculation or contact challenge of probang sampling, first letter corresponds to VI result, second letter corresponds to rRT-PCR result. P: positive, N: negative

### Antemortem sample collection and processing

Antemortem sampling consisted of collection of sputum samples using a probang cup as described in the OIE Terrestrial Manual 2012, Chapter 2.1.5, section B [2]. Briefly, approximately 10 ml of OPF was collected with probang cups and transferred to ice-cooled conical tubes containing 10 ml of MEM with 25mM Hepes. OPF samples were frozen in 1–2 hours to -70°C until further processing (see below).

### Postmortem sample collection and processing

Necropsies were performed immediately subsequent to euthanasia at predetermined timepoints. Sample collection schemes were predetermined and standardized with minor variation among individual animals. A maximum of 28 anatomically distinct tissues were collected per animal. Tissue specimens were collected from the oral cavity, nasal cavity, soft palate, pharynx, larynx, trachea, lungs, lymph nodes and skin (Table 2) as previously described [35]. For each defined specimen, two 30 mg tissue samples were aliquoted into separate screw-cap 1.5 ml cryovials and frozen immediately in liquid nitrogen for transfer within 2 h to a -70°C freezer in which they were stored until the time of processing. An adjacent specimen from each tissue was placed in a cryomold, embedded in Optimal Cutting Temperature Compound (OCT) (Sakura Finetek, Torrance, CA), frozen on a bath of liquid nitrogen, and stored at—70°C for immunomicroscopy.

**Table 2:** Postmortem results of FMDV detection in 21 FMDV persistently infected cattle.

Strain	A24 RGD							A24 SGD							O1 Manisa			SAT2						
Steer#	392	393	395	242	400	020	244	233	755	560	207	199	004	030	626	625	624	612	613	614	618	Cumulative Percentage Positivity		
Route of inoculation	IDL ^a^	IDL	CT^b^	IDL	IDL	CT	CT	IDL	IDL	IDL	CT	V/C^c^	V/I^d^	V/I	CT	IDL	IDL	V/I	V/I	V/I	IDL			
Necropsy performed at DPI ^e^	41	41	41	35	35	36	36	28	28	29	44	44	42	42	37	37	40	44	44	45	52	rRT-PCR ^f^	VI ^g^	rRT-PCR or VI
Oral Cavity / Oropharynx																								
Tongue (rostral)	neg ^h^	neg	neg	NE ^i^	NE	NE	NE	NE	NE	NE	neg	neg	neg	neg	NE	NE	neg	neg	neg	neg	neg	0.00	0.00	0.00
Dental Pad	neg	NE	NE	NE	NE	NE	NE	NE	NE	NE	NE	NE	NE	NE	NE	NE	neg	neg	neg	neg	neg	0.00	0.00	0.00
Ventral Soft Palate (rostral)	neg	neg	neg	neg	neg	neg	neg	3.49^j^	neg	neg	neg	neg	NE	NE	neg	neg	neg	neg	neg	neg	neg	5.26	0.00	5.26
Ventral Soft Palate (caudal)	neg	neg	4.33	neg	neg	neg	neg	neg	3.00	neg	neg	neg	neg	neg	2.35	neg	neg	2.77	neg	neg	neg	19.05	0.00	19.05
Nasopharynx / Larynx																								
Dorsal Soft Palate (rostral)	**4.29** ^k^	neg	**5.04**	4.80	neg	2.90	3.87	**3.38**	**neg**	**3.50**	2.50	neg	neg	neg	neg	**neg**	neg	4.81	2.72	neg	neg	47.62	28.57	57.14
Dorsal Soft Palate (caudal)	2.52	**3.82**	**2.69**	neg	neg	neg	3.52	**2.50**	**neg**	**neg**	2.93	4.75	neg	neg	**2.29**	**neg**	2.28	neg	3.35	2.54	3.05	57.14	33.33	71.43
Dorsal Nasopharynx (rostral)	3.70	neg	4.13	**neg**	3.43	neg	2.36	**4.18**	**4.86**	**3.67**	**neg**	**5.75**	neg	**3.63**	**2.66**	**3.88**	neg	4.01	3.92	2.32	3.14	71.43	42.86	80.95
Dorsal Nasopharynx (caudal)	3.30	neg	4.31	neg	neg	neg	2.44	4.10	**2.34**	**neg**	2.61	2.65	neg	3.16	**2.85**	neg	neg	2.44	3.07	neg	neg	52.38	14.29	57.14
Epiglotis	5.17	**3.35**	**3.71**	3.09	neg	**3.33**	5.52	neg	NE	**3.94**	3.92	4.82	neg	neg	**3.14**	**neg**	neg	neg	3.94	neg	3.46	60.00	30.00	65.00
Ventral Larynx	4.75	neg	neg	neg	neg	neg	NE	neg	neg	3.18	neg	3.51	neg	neg	**neg**	neg	neg	neg	neg	neg	neg	15.00	5.00	20.00
Lungs / Trachea																								
Trachea 10 cm	neg	neg	neg	NE	NE	NE	NE	NE	NE	NE	NE	NE	NE	NE	NE	NE	neg	neg	neg	neg	neg	0.00	0.00	0.00
Bronchial Bifurcation	neg	NE	NE	NE	NE	NE	NE	NE	NE	NE	NE	NE	NE	NE	NE	NE	neg	neg	neg	neg	neg	0.00	0.00	0.00
Distal Cranial Lobe	neg	NE	NE	NE	NE	NE	NE	NE	NE	NE	neg	neg	NE	NE	NE	NE	neg	neg	neg	neg	neg	0.00	0.00	0.00
Distal. mid. Lobe	neg	neg	neg	NE	NE	NE	NE	NE	NE	NE	neg	neg	neg	neg	NE	NE	neg	neg	neg	neg	neg	0.00	0.00	0.00
Distal Caudal Lobe	neg	NE	NE	NE	NE	NE	NE	NE	NE	NE	neg	neg	NE	NE	NE	NE	neg	neg	neg	neg	neg	0.00	0.00	0.00
Tonsils / Lymph Nodes																								
Nasopharyngeal Tonsil	neg	neg	neg	neg	neg	neg	neg	**4.77**	neg	neg	2.42	neg	neg	neg	neg	neg	neg	neg	neg	neg	neg	9.52	4.76	9.52
Palatine tonsil	neg	neg	neg	neg	neg	2.40	neg	neg	neg	neg	neg	2.83	neg	neg	neg	neg	neg	neg	neg	neg	neg	9.52	0.00	9.52
Lingual tonsil	neg	neg	neg	NE	NE	NE	NE	NE	NE	NE	neg	neg	neg	neg	NE	NE	neg	neg	neg	neg	neg	0.00	0.00	0.00
Retropharyngeal LN ^l^	neg	neg	2.67	2.30	neg	neg	NE	neg	neg	3.20	2.69	2.67	neg	2.88	neg	neg	neg	neg	neg	neg	neg	30.00	0.00	30.00
Submandibular LN	neg	neg	neg	neg	neg	2.60	3.93	2.24	3.74	neg	2.59	neg	neg	neg	neg	neg	neg	neg	neg	neg	neg	23.81	0.00	23.81
Parotid LN	neg	NE	NE	NE	NE	NE	NE	NE	NE	NE	NE	NE	NE	NE	NE	NE	neg	neg	neg	neg	neg	0.00	0.00	0.00
Hilar LN	neg	neg	neg	neg	neg	neg	neg	neg	neg	neg	neg	neg	neg	neg	neg	neg	neg	neg	neg	neg	neg	0.00	0.00	0.00
Additional Tissues																								
Esophagus	NE	neg	NE	NE	NE	NE	neg	NE	NE	NE	NE	NE	neg	neg	NE	NE	NE	NE	NE	NE	NE	0.00	0.00	0.00
Nasal Septum	neg	NE	NE	NE	NE	NE	NE	NE	NE	NE	NE	NE	NE	NE	NE	NE	neg	neg	neg	neg	neg	0.00	0.00	0.00
Parotid gland	neg	NE	NE	NE	NE	NE	NE	NE	NE	NE	neg	neg	NE	NE	NE	NE	neg	neg	neg	neg	neg	0.00	0.00	0.00
Interdigital Cleft (RF)	NE	neg	neg	NE	NE	NE	NE	NE	NE	NE	neg	neg	NE	NE	NE	NE	NE	NE	NE	NE	NE	0.00	0.00	0.00
Coronary Band	NE	NE	NE	NE	NE	NE	NE	NE	NE	NE	neg	neg	NE	NE	NE	NE	NE	NE	NE	NE	NE	0.00	0.00	0.00
Metacarpal Skin	NE	NE	NE	NE	NE	NE	NE	NE	NE	NE	neg	neg	NE	NE	NE	NE	NE	NE	NE	NE	NE	0.00	0.00	0.00

^a^ IDL: intradermolingual (direct inoculated)

^b^ CT: contact inoculated

^c^ V/C: Vaccinated and Challenged by Direct Contact

^d^ V/I: Vaccinated and Challenged by Intradermolingual Inoculation

^e^ DPI: Days Post Inoculation

^f^ rRT-PCR: real-time Reverse Transcriptase Polymerase Chain Reaction

^g^ VI: Viral Isolation; results obtained will fall into two categories, positive or negative

^h^ neg: Negative

^i^ NE: Not Evaluated

^j^ log10 FMDV RNA copy numbers per mg of tissue

^k^ bold font indicates virus isolation positive samples.

^l^ LN: Lymph nodes

### Foot and mouth disease virus RNA detection

For antemortem samples (probangs), rRT-PCR was performed as described below without any other treatment. For postmortem samples (tissues), two specimens of each tissue listed in Table 2 were thawed and immediately macerated in a TissueLyser bead beater (Qiagen, Valencia, CA) as previously described [35]. For RNA extraction of probang samples and macerated tissues, 50 μl of each sample was transferred to a 96-well plate (Thermo Scientific, Waltham, MA) containing 150 μl lysis/binding solution. RNA was subsequently extracted using Ambion’s MagMax-96 Viral RNA Isolation Kit (Ambion, Austin, TX) on a King Fisher-96 Magnetic Particle Processor (Thermo Scientific, Waltham, MA). RNA was eluted in a final volume of 25 μl. Once extracted, 2.5 μl of RNA was analyzed by rRT-PCR on the ABI 7000 system (Applied Biosystems, Austin, TX) as previously described [35]. Samples with cycle threshold (Ct) values < 40 were considered positive. rRT-PCR results were converted to FMDV RNA copy numbers per mg of tissue as previously described [36]. The Ct positivity cutoff of 40 corresponded to a detection threshold value of 2.24 log_10_ FMDV RNA copies/mg (FMDV RNA/mg) of tissue. Real-time rRT-PCR results reported in Table 2 are the higher FMDV RNA/mg value of the two samples processed per tissue per animal.

### Foot-and-mouth disease virus isolation

Tissues obtained postmortem were macerated as described above and cleared of bacterial contamination using centrifuge tube filters (Spin-X, Costar, Corning, NY). In order to dissociate virus-antibody complexes and thereby improve infectious virus detection, probang fluid samples were treated with TTE [37]. For this purpose, 2 ml of probang and 2 ml of TTE were homogenized in 6 ml tubes using the Tissues Lyser described above. Samples were then clarified by centrifugation at 1000 rpm for 2 min at 4°C and the supernatant was cleared of bacterial contamination using centrifuge tube filters (Spin-X, Costar, Corning, NY). VI was performed as previously described [35], separately on the duplicate samples of each tissue or probang sample on LFBK cells expressing the bovine αvβ6 integrin [38]. Upon detection of cytopathic effect (CPE), FMDV positivity was confirmed by rRT-PCR on cell culture supernatants. Samples in which no CPE was observed were amplified through three blind passages and the supernatants tested by rRT-PCR before they were deemed negative. VI results in Table 2 are reported positive if either or both duplicate samples per tissue were positive.

### Tissue-specific distribution of FMDV and viral RNA

Tissue—specific, cumulative positivity percentages (PP) were calculated for each tissue for rRT-PCR, VI, and combined rRT-PCR or VI. The PP was defined as: *total positive results at tissue X by modality Y in persistent steers / total specimens of tissue X examined by modality Y in persistent steers*. Thus, PP served as an indicator of the prevalence of involvement of each tissue in persistent FMD in these animals. Within each testing modality (rRT-PCR, VI, rRT-PCR or VI), PP values were statistically compared across the stratifying variable “tissue category” by analysis of variance (ANOVA) in commercially available software (Microsoft Excel 2007).

### Host IFN & ISG mRNA detection and analyses

Aliquots of 30 mg of tissues were individually lysed by adding 600μL of RLT lysis buffer (Qiagen, Valencia, CA) and macerated using the rotor-stator method (TissueMizer, Fisher Scientific). Approximately 600μl of homogenate was transferred to a Qiagen Qiashredder (Qiagen), and total RNA was subsequently isolated using an RNeasy kit (Qiagen) as recommended by the manufacturer. RNA concentrations were determined using a NanoDrop ND-1000 spectrophotometer. 1.0μg of RNA was treated with DNase I per manufacturer’s instructions (Sigma, St. Louis, MO.) and total RNA was reverse transcribed into cDNA using random hexamers (Thermo Scientific Hanover Park, IL.) per manufacturer’s instructions. Briefly, the 25μL reactions contained 11.0μL of DNAsed RNA, 5.0μL of 5x First-strand buffer (250mM Tris HCl, 375mM KCl, 15mM MgCl2), 2.5μL of 0.1M DTT, 2.5μL of random hexamers (125ng/μL), 1.25μL of RNAseOut Recombinant Ribonuclease Inhibitor (Invitrogen #10777–019), 1.0μL (200units) of Moloney murine leukemia virus reverse transcriptase (Invitrogen #28025–013), 0.125mM deoxynucleoside triphosphates (Applied Biosystems #N808-0007), and 0.5μL H20. Samples were thermocycled at 25°C for 10 min, 37°C for 60 min, and 95°C for 5 min. The cDNA was then diluted with distilled water 1:8 in a final volume of 200μl.

Cytokine rPCR was carried out on the ABI 7000 Sequence Detection System. Briefly, the 25.0μL reactions contained 12.5μL Taqman 2x PCR Master Mix (Applied Biosystems #4304437), 300nM final concentration of each primer (Invitrogen), 150nM final concentration of Taqman 6FAM-labeled fluorogenic probe (Applied Biosystems), 4.5μL H2O, and 2.0μL of cDNA template. Samples were thermocycled at 50°C for 2 minutes, 95°C for 10 minutes, and 40 cycles of 95°C for 15 seconds and 60°C for 60 seconds.

Baseline levels of expression of 14 host genes of interest (TNF-α, IFN-α, IFN-β, IL-1β, IL-2, IL-4, IL-6, IL-10, IL-12, IL-15, IL-18, IFN-γ, IP-10 and RANTES) were established from caudal DSP of mock-inoculated steers (n = 3) by rPCR systems as previously described [21]. cDNA was generated and rPCR was performed as described above. cDNA was run in triplicate and averaged for each individual specimen. The triplicate averages of the 3 steers were then averaged to generate the negative control baseline Ct for each tissue for all 14 genes of interest. Tissues (i.e. caudal DSP from 12 FMDV-infected steers) were collected, processed, and analyzed for expression of the 14 genes of interest as described above.

### Analysis of cytokines gene expression in FMD carrier animals- real time PCR statistical analysis

Cytokine gene expressions of caudal DSP tissue samples, collected from 12 FMDV-carrier and 3 control (uninfected) animals were analyzed. Relative gene expression between FMDV-carriers and controls was normalized using the GAPDH housekeeping gene. The statistical comparison between the carrier and control groups was carried out by randomizing data points from each group and calculating the relative expression [39]:

$\frac{{E_{Ck}}^{\Delta {Ct}_{Ck}(control-carrier)}}{{E_{GAPDH}}^{\Delta {Ct}_{GDPH}(control-carrier)}}$, (formula 1) where E is the real-time—PCR assay, Ck refers to each of the cytokines evaluated, and the ∆Ct is the cycle difference between the RNA detection in the control and the carrier sample.

Randomizations and bootstrapping methods to compute statistical significance and confidence intervals were calculated using REST 2009© software [40], generating 6000 random pairs of carriers and control, and using assay efficiency previously determined for each assay in our laboratory.

### Association of cytokines relative expression and virus isolation or real-time PCR detection in carrier animals

The relative expression ratio of cytokines for each animal was computed using formula 1, where Ct values for individual carrier animals and the mean Ct values of the three control animals. Animals were categorized in two groups, based on the relative expression ratio value >or <1 (up or down regulation of gene expression). The association of cytokine expression and the VI or PCR test outcome was assessed using a Chi^2^ test. SPSS software was used to compute Chi^2^ values.

### Immunomicroscopy

Microscopic localization of FMDV antigens and host proteins was performed in cryosections as previously described [41]. For immunohistochemistry, detection was performed with micropolymer alkaline phosphatase kit (Biocare Medical). For multichannel immunofluorescence (MIF), detection was performed with goat anti-mouse isotype-specific secondary antibodies labeled with AlexaFluor dyes (AF—405, 488, 594, 647). The mouse monoclonal antibodies used for detection of FMDV-VP1 and -3D proteins have been described previously [42,43]. Antibodies used to label cell markers in MIF experiments were mouse monoclonal anti-pancytokeratin plus (Biocare #CM162), anti-MHCII (AbD Serotec, MCA2225PE), and anti-CD11c (VMRD Clone BAQ153A). For each MIF experiment, a duplicate, negative-control serial section treated with an isotype-matched irrelevant antibody or isotype control reagent of similar concentration was included.

## Results

### Determination and characterization of persistent infections

Twenty one of 46 animals (45.7%) had FMDV in OPF or tissues on or after 28 dpi and thereby met the required definition of FMDV persistence (Tables 1 and 2). For each selected animal, at least one or as many as 12 probang samples tested positive for FMDV and/or FMDV RNA. FMDV or viral RNA was recovered from OPF of eighteen steers within the two days preceding euthanasia. Due to logistical constraints, three animals were euthanized later at four days (#004, #030) or five days (#618) after a positive OPF sample (Table 1). Most probang samples collected from these 21 selected carrier animals were positive by rRT-PCR or by virus isolation (97.22%), with 91.66% positive for rRT-PCR, 81.48% positive for VI and 75.92% positive by both techniques. Interestingly, only amongst SAT2-infected animals, several probangs were found to contain infectious FMDV, but not viral RNA.

### Tissue-specific distribution of FMDV and viral RNA in persistent cattle

**Fig 1:**
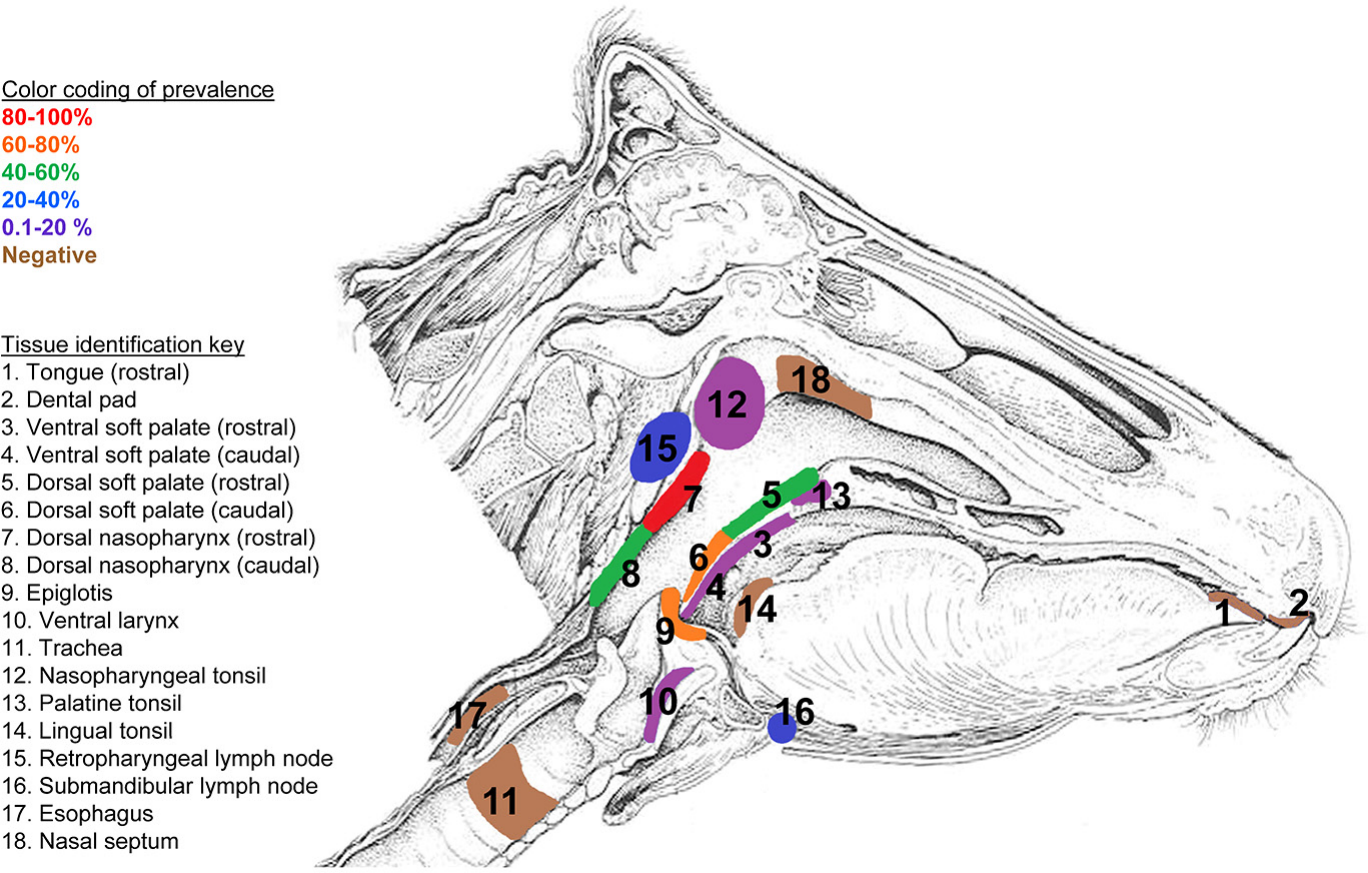
Tissue-specific distribution of FMDV. Detection was performed by virus isolation or rRT-PCR in persistently infected steers inoculated by direct (intradermolingual route) or contact exposure inoculation. Only the epithelium of the Dorsal Nasopharynx (7) occupies the highest stratum of 80–100% indicating this tissue as the most consistent site of FMDV persistent infection. Prevalence values were calculated as number of animals in which a tissue was determined positive by one or both techniques/total number of animals.

Tissue—specific, cumulative percentages positivity (PP) were calculated for detection of FMDV by rRT-PCR, VI, and combined rRT-PCR or VI for animals spanning all three viral strains (Fig 1 and Table 2). Thus, PP served as an indicator of the prevalence of involvement of each tissue in FMDV persistence. Amongst all tissues examined, the nasopharynx/larynx region contained the sites with the highest PP for infectious FMDV and FMDV RNA. The highest PP value for detection of FMDV RNA (71.43%), infectious virus (42.86%) and combined FMDV detection (80.95%) occurred in the rostral dorsal nasopharynx. The tissues with next highest combined PP values were caudal dorsal soft palate (71.43%) and epiglottis (65.00%). PP values for rRT-PCR, VI, rRT-PCR or VI were each significantly higher for the category “nasopharyngeal/laryngeal” tissues compared to all other tissues (p< 0.001). Within the “nasopharyngeal/laryngeal” tissues, mean FMDV RNA genome copy number was higher amongst nasopharyngeal tissues compared to tissues from the larynx; however this difference was not statistically significant. Neither infectious virus nor FMDV RNA was ever detected in tongue, dental pad, nasal septum, trachea, bronchial bifurcation, lungs, parotid and hilar lymph nodes, esophagus, parotid salivary gland and skin (interdigital cleft, coronary band and metacarpal skin) (Fig 1 and Table 2).

### Cytokine mRNA expression

**Fig 2:**
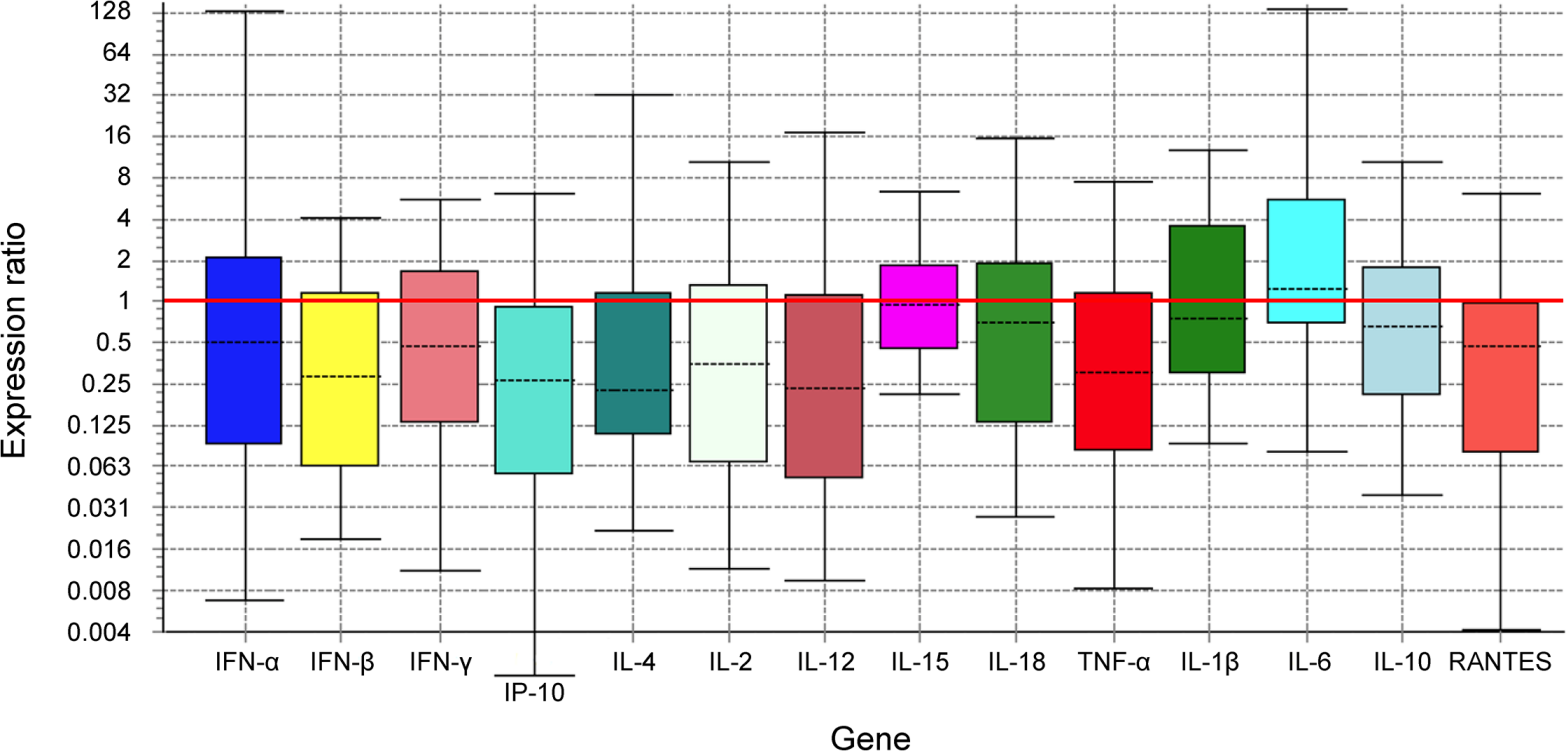
Analysis of relative expression of different cytokines in dorsal soft palate of persistently infected steers. Boxes represent the interquartile range, or the middle 50% of observations. The dotted line represents the median gene expression. Whiskers represent the minimum and maximum observations.

Relative mRNA expression of a number of cytokines of interest were measured in caudal DSP tissue with means compared between 12 FMDV-persistently infected steers and three naïve steers. This comparison tested the null hypothesis that the FMDV carrier state is not associated with any alteration of transcription of cytokine genes. Although no statistically significant differences in cytokine mRNA transcription between carriers and uninfected control animals were observed, there was a general trend of reduction in the expression in tissues of carriers as compared to uninfected controls for most of the cytokines examined (Fig 2, Table 3). This is reflected by mean expression ratios weighted below 1.0 for 12 out of 14 cytokines. Specifically, amongst carriers there was 2-fold or more reduction of IFN-α, IFN-β, IFN-γ, IP-10, IL-2, IL-4, IL-12, TNF-α and RANTES. Cytokines IL-10, IL-15 and IL-18 had a reduction of expression in between 1 and 2-fold and IL-1β had no detectable change in expression (expression ratio = 1). IL-6 was the only cytokine showing increased expression amongst carriers with more than two-fold increase of relative expression.

**Table 3:** Results of mean relative expression ratio and confidence intervals for each cytokine in all animals tested.

Gene	Type	Reaction Efficiency	Expression	Std. Error	95% C.I.	P(H1)*
GAPDH	reference	0.92	1.000			
IFN-α	target	1.00	0.500	0,064–7,221	0,011–115,863	0,601
IFN-β	target	0.93	0.269	0,046–1,490	0,021–2,482	0,195
IFN-γ	target	0.99	0.413	0,060–2,004	0,028–4,528	0,381
IP-10	target	0.97	0.198	0,025–1,208	0,003–3,198	0,177
IL-2	target	1.00	0.312	0,042–2,049	0,016–5,141	0,306
IL-4	target	1.00	0.355	0,076–2,584	0,038–25,411	0,332
IL-12	target	1.00	0.275	0,038–2,042	0,010–11,822	0,348
IL-15	target	0.94	0.934	0,358–2,394	0,227–4,151	0,839
IL-18	target	1.00	0.539	0,054–3,421	0,031–12,813	0,593
TNF-α	target	1.00	0.316	0,052–2,150	0,020–5,606	0,293
IL-1β	target	0.97	1.011	0,185–5,550	0,112–10,365	0,994
IL-6	target	0.98	2.065	0,489–7,951	0,101–115,755	0,546
IL-10	target	0.82	0.643	0,131–2,967	0,043–9,601	0,636
RANTES	target	0.95	0.318	0,045–1,751	0,007–4,141	0,290

For all cytokines examined, sample (carrier) group is not significantly different from control (baseline) group.

* P(H1)—Probability of alternate hypothesis that difference between sample and control groups is due only to chance.

### Microscopic Localization of FMDV Antigens and Phenotypic Characterizations of Associated Cells

**Fig 3:**
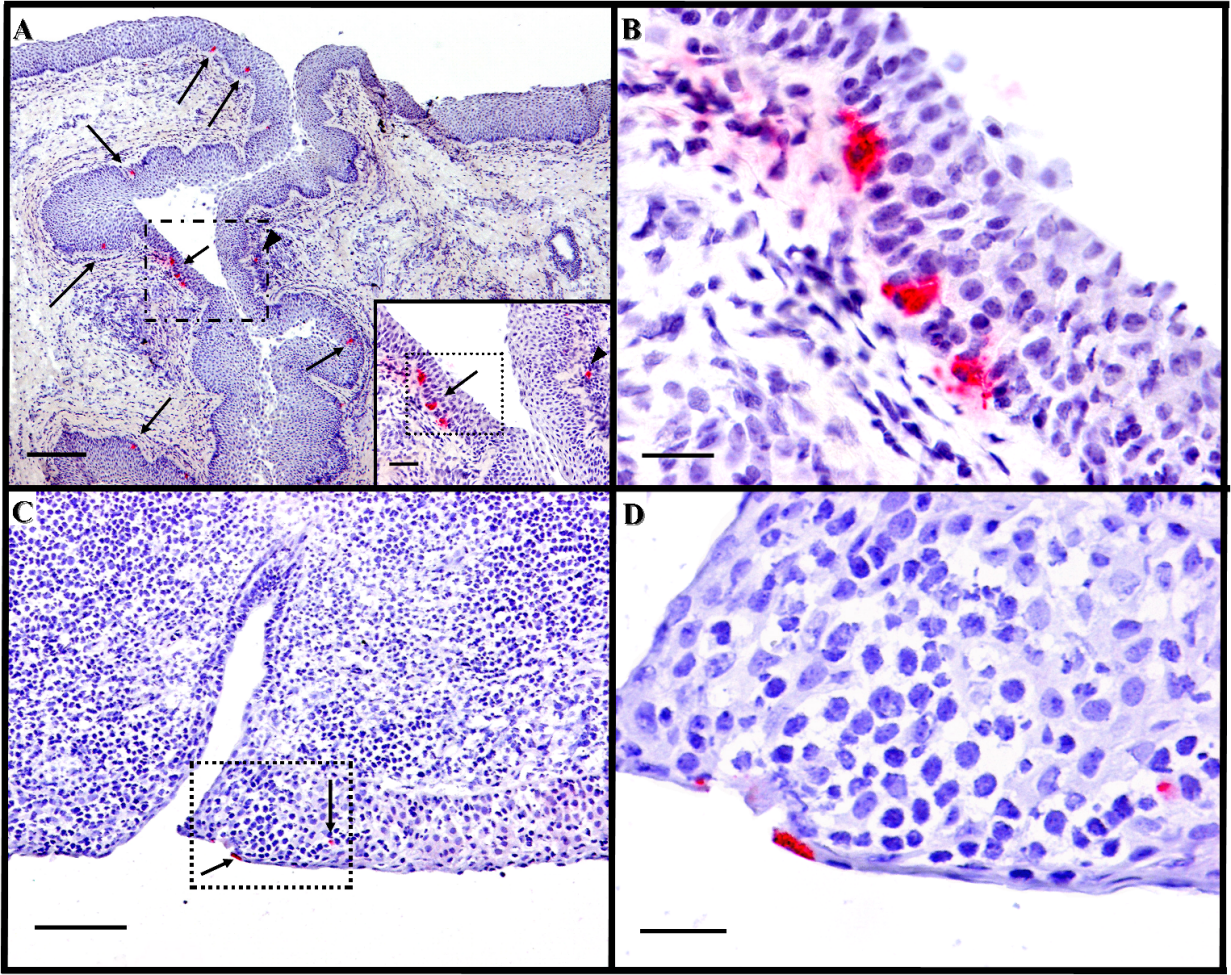
Immunohistochemical detection of persistent FMDV in nasopharyngeal mucosa. **A**) Dorsal soft palate (caudal), steer #626, 37 dpe, FMDV O1 Manisa. FMDV non-structural protein localized to multiple cells within basal layers of crypt epithelium (arrows) as well as a single cell within associated lymphoid follicle (arrow head). 4x magnification, scale bar 200μm. Anti-FMDV 3D monoclonal antibody. Micropolymer alkaline phosphatase. Gill s hematoxylin counterstain. Inset; 20x magnification of region indicated in dashed rectangle in A, scale bar 50μm. **B)**, 40x magnification of region within dashed rectangle in Fig 3A inset. Large, polygonal, immunopositive cells are within basal epithelium, scale bar 25 μm. **C)** Dorsal nasopharynx (rostral) steer #625, 37 dpi, FMDV O1 Manisa. FMDV structural protein localized to scarce individual cells within superficial and deeper layers of MALT-associated surface epithelium (arrows). 10x magnification, scale bar 100 μm. Anti-FMDV VP1 monoclonal antibody. Micropolymer alkaline phosphatase. Gill’s hematoxylin counterstain. **D**) 40x magnification of region within dashed rectangle in Fig 3C, superficial cells containing FMDV antigen are squamous, whereas deeper immunopositive cell is polygonal, scale bar 25 μm.

**Fig 4:**
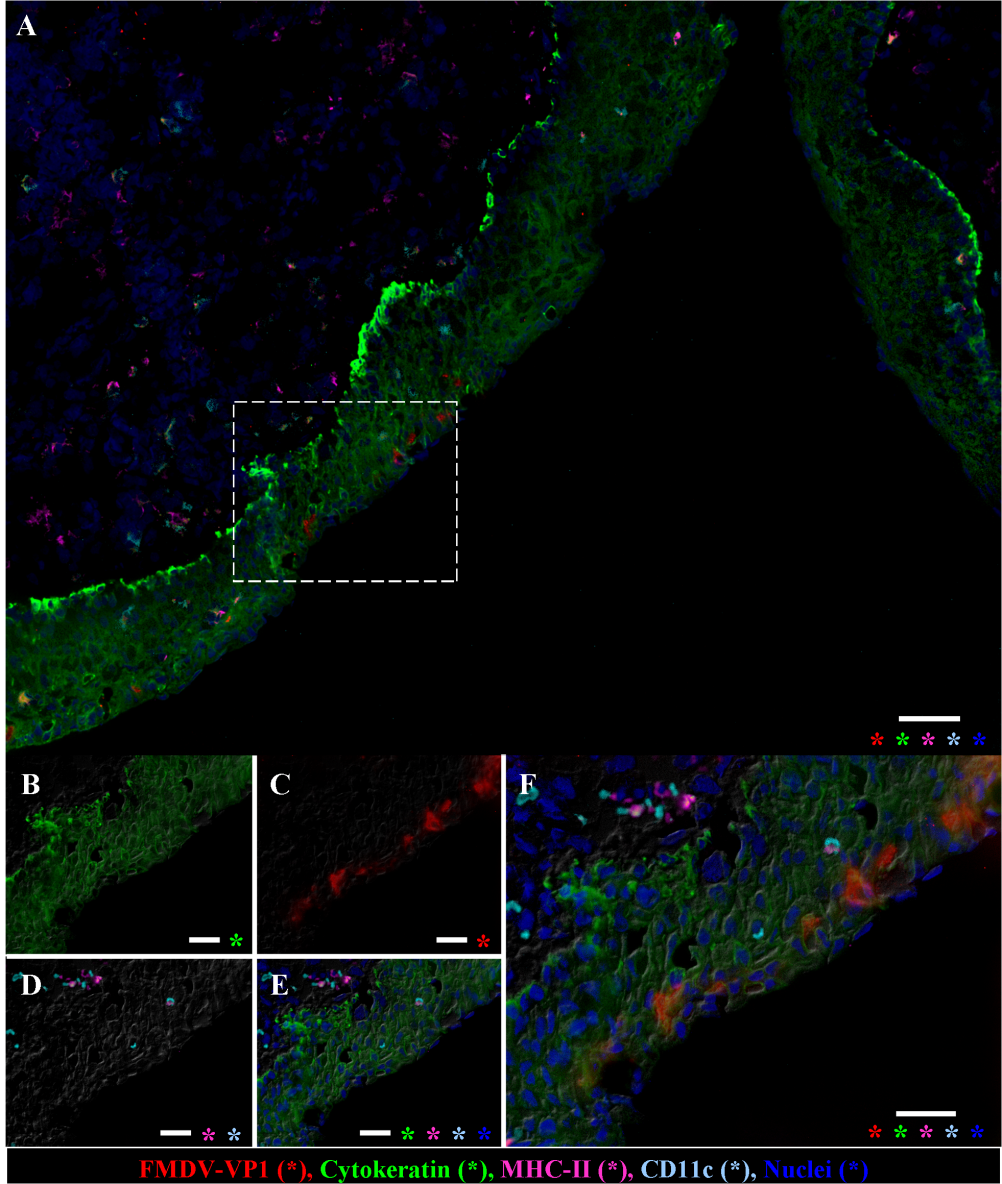
Immunofluorescent detection of persistent FMDV in nasopharyngeal mucosa. Dorsal nasopharynx, steer #626, 37dpe, FMDV O1 Manisa. Immunomicroscopy, **A)** Low magnification (10X) view of an epithelial invagination with a focal cluster of FMDV-antigen-positive cells within superficial epithelial surface, scale bar 50 μm. **B-F)** Higher magnification (40X) views of region of interest identified in A (dashed box). Cells containing FMDV-VP1 are in the superficial epithelium and are cytokeratin-positive. Few MHC-II and CD11c positive cells are present within and below epithelium, but do not contain FMDV-VP1. Indirect fluorescence technique with differential interference contrast, antibody labels color-coded in bottom panel, asterisks indicate channels present in each panel, scale bar 25 μm.

Tissues within which FMDV or FMDV RNA were detected were further examined by conventional microscopy, immunohistochemistry (Fig 3) and MIF microscopy (Fig 4) with anti-FMDV-VP1 (capsid) and anti-FMDV-3D (non-structural) primary antibodies. In all cases, epithelia were consistently intact with no evidence of erosion, ulceration or microvesiculation. Lymph nodes and MALT were populated by appropriate quantity of cells with normal tissue architecture. FMDV antigen was microscopically localized within a subset of analyzed tissues. Within nasopharyngeal epithelia, FMDV structural and non-structural proteins were identified by immunohistochemistry as scarce individual cells or as small clusters of up to10 immunopositive cells within basal and/or superficial layers of epithelium (Figs 3 and 4). Epithelial segments containing FMDV antigen were typically closely associated with MALT follicles and were often lining the opening or lumens of crypts. Additionally, rare single cells containing FMDV protein were localized to subepithelial lymphoid regions. Simultaneous immunostaining for host cell antigens indicated that FMDV-positive cells were morphologically and phenotypically consistent with epithelial cells (cytokeratin+/MHCII^-^/CD11c^-^; Fig 4). In some tissues, cytokeratin^-^/MHCII+ intra-epithelial cells (presumptive DC) were in close proximity, but were never observed to contain FMDV antigen.

## Discussion

In order to study virus-host interactions in persistent FMD in cattle, carrier steers were investigated for tissue-specific virus distribution and host responses. Although it is thoroughly established that carrier cattle shed FMDV into OPF, there is some discrepancy regarding the precise anatomic site(s) of virus persistence. Earlier studies have reported that the epithelial cells of pharyngeal tissues [16,17,44] and/or retropharyngeal and submandibular lymph nodes [15,44] are the predilection site for FMDV persistence in cattle. This consensus is challenged by a recent study that suggests that the DSP and the submandibular and retropharyngeal lymph nodes cannot be definitively concluded to be the principal sites for persistence of FMDV due to inconsistent detection of FMDV RNA in these tissues [22]. The extent to which these differences across studies reflect variation in experimental designs, utilization of different FMDV strains, different treatments before inoculation, as well as different inoculation routes remains undetermined.

In the current study we confirmed that the critical and consistent anatomic regions involved in persistent FMDV infection in cattle are within the nasopharynx and larynx. This was accomplished through examination of up to 28 distinct tissues per animal from 21carrier cattle infected with one of three serotypes of FMDV. Specifically, dorsal nasopharynx, dorsal soft palate, and epiglottis had the greatest proportional detection of FMDV and FMDV RNA across all viruses. Interestingly, the tissues with the highest prevalence were located with tridimensional contiguity, forming the floor and roof of the nasopharynx. This anatomical region within Waldeyer’s ring [45] has been reported to include primary sites of FMDV infection in cattle [22,26,35,36,46–50]. Thus, these pharyngeal tissues have unique structural and/or functional properties that facilitate permissiveness to FMDV infection and maintenance during early and persistent phases of FMD. FMDV and FMDV RNA were also detected within ventral soft palate, ventral larynx, regional lymph nodes (retropharyngeal and submandibular) and tonsils (palatine and nasopharyngeal) at lower prevalence. These findings are consistent with previous studies [14,51] and suggest that these tissues also may be involved in FMDV persistence, but with less consistency than the pharyngeal epithelia (MALT).

Microscopic examination of numerous FMDV-positive nasopharyngeal tissues from confirmed carrier cattle indicated that no histopathological lesions were present in any animal. This suggests that the interaction of virus and host in the nasopharynx during the carrier state is quite different from what occurs during acute infection. The characteristic vesicles of feet and mouth, for which FMD is named, have classical microscopic lesions consisting of acantholytic degeneration of keratinocytes, epithelial spongiosis, and sloughing of affected regions with resultant erosions [41,52]. Primary infection of the nasopharyngeal epithelium of cattle induces a less severe extent of an apparently similar process resulting in keratinocyte degeneration and ultimately cell loss (erosion) [36]. By contrast, the nasopharyngeal epithelium of carriers was consistently found to be intact with no evidence of erosion even in areas in which FMDV antigen was localized. This lack of evidence of cellular degeneration of FMDV-infected cells in carriers suggests a distinct mechanism in which infected cells survive and remain within the carrier’s epithelium. These findings are consistent with previously published suggestion that in the carrier state, FMDV employs a distinct, non-cytolytic infection process which may be driven by alterations in cytokine signaling cascades and/or tissue specific decreased cell death mechanisms [3,53].

FMDV antigens were rarely microscopically localized in tissues which (macroscopically) contained FMDV and/or FMDV RNA. In regions where antigen was found, it was localized to foci of individual, or small clusters of, immunopositive cells. This contrasts previous reports that have demonstrated regionally diffuse localization of FMDV RNA by in situ hybridization in the pharynx of carrier cattle [16,17]. The reason for this difference in localization is unclear, but may further support the concept of a limited virus replication cycle in carriers whereby only a subset of FMDV RNA-containing cells progress to producing viral antigens. The phenotypic profile of intra-epithelial cells containing FMDV antigens was uniformly cytokeratin (+)/ CD11c(-)/MHCII(-). This suggests that replication of FMDV in the pharyngeal epithelium of carrier cattle occurs in epithelial cells rather than intra-epithelial dendritic (Langerhans-like) cells.

The current data suggest that in the persistent phase of FMDV infection in cattle there is a clear lack of stimulation and a trend towards suppression of transcription of various host cytokine genes at the sites of virus persistence. Recent reports from our laboratory describing the acute phase of FMD in cattle demonstrated modest induction of some of the same cytokine genes at these same tissues [26] and tissue-specific induction of IFN signaling pathways [53]. In the acute phase of infection, FMDV is largely similar to several other viral infections that generally induce proinflammatory and antiviral innate immune responses [13,26,28,53–56]. By contrast, there are examples wherein the persistent phase of FMDV and other viral infections have been shown to be associated with suppression or modulation the host immune response [13,57,58]. Thus, our demonstration of relative suppression of transcription of IFN-α/β, TNF-α, IL-12 and RANTES has some similarity to the suppressing expression of proinflammatory cytokines observed in herpes simplex virus type 1 during persistence [59]. However, no conclusive reports of immune system gene suppression have been published associated with FMDV persistent infection. Indeed, two studies have demonstrated upregulation of TNF-α in tissues of cattle persistently infected with FMDV [13,34]. Additionally, decreased detection of IFN-β and lower IFN signaling capacity have been demonstrated during the acute phase of FMDV infection of cattle [13,53].

There are other comparisons that may be drawn between the cytokine modulation described herein and previous accounts describing other viral infections. IL-12, suppressed in the current study, has previously been shown to be inhibited by Measles virus and human immunodeficiency virus (HIV), and thereby demonstrated to subvert the development of cell-mediated immunity [60]. Bovine rotavirus and bovine coronavirus have been demonstrated to downregulate IFN and pro-inflammatory cytokine-associated pathways [54]. Additionally, persistent hepatitis C virus infection has been associated with disruption of the cellular signaling pathways which lead to interferon production, thus blocking antiviral activities of ISGs to evade innate immunity, contributing to virus persistence and resistance to therapy [55].

The establishment of persistent FMDV infection provides an example of a classic pathogenesis battle in which the pathogen achieves victory over the defeated host immune response. However, the mechanism whereby FMDV evades the host response is still unclear. Considering the current transcriptome data in the context of previously published works allows some novel insights into the mechanisms that contributed to this process. It is well established that FMDV is highly sensitive to IFNs type I, II, and III [61–67] and several studies have documented IFN production by cattle as part of the acute response to FMDV infection [13,26,68]. Thus, the decreased relative expression of mRNAs for IFN-α, -β and -γ in pharyngeal tissues of the FMDV carriers described herein suggests an association between transcription suppression of these genes and the FMDV carrier state. Specifically, our findings of a 3 to 4-fold suppression of IFN-β and TNF-α mRNA expression amongst carriers suggests immunomodulation that may benefit the persistence of FMDV as has been suggested by other investigators [13,34]. However, the causality of this association remains undermined; specifically, one might consider whether 1) FMDV dysregulated the expression of these genes thus enabling the carrier state or 2) a subset of cattle are intrinsically poor producers of IFN and it is these animals that ultimately become carriers. Previous work describing gene expression in tissues with different FMDV tropism from non-infected cattle suggested that pharyngeal tissues are intrinsically poor type I and III IFN inducers and responders, and this might explain their susceptibility to both primary and persistent infection (Zhu et al 2013). The results of the current study are consistent with those findings.

The manners in which innate cytokine production influence pathways of cellular immunity add another level of mechanistic complexity to considering transcriptome analysis. Specifically, in the current study IFN-γ mRNA was decreased more than 2-fold among carrier animals. IFN-γ induces the production of interferon gamma-induced protein 10 (IP-10), which together with IL-4 is involved in epidermal DC activation, maturation and function [69,70]. Mature DC are able to produce IL-2, IL-12, IL-15 and IL-18 and these cytokines are involved in NK cell recruitment, proliferation and activation that would ultimately eliminate FMDV infected cells (as reviewed by Toka *et al* [71]). In the current report, we have documented more than 2-fold suppression of IP-10 and IL-4, as well as IL-2 and IL-12, up to 2-fold suppression for IL-18, and no substantial effect on IL-15 in the persistently infected tissues of carriers. Thus, these alterations of regional cytokine microenvironment may preclude the cellular pathways that are required to eliminate FMDV infected cells.

## Conclusion

The current work has contributed to further defining the anatomical sites and host processes associated with persistence of FMDV in cattle. Trimodal detection of infectious FMDV, FMDV RNA, and viral antigen has confirmed the importance of the nasopharyngeal mucosal surfaces as sites of FMDV persistence. Although it is well-established that FMDV has immunomodulatory effects at various tissues at various stages of infection, precise understanding of these processes remains elusive. Through comparison of cytokine transcription levels in persistently infected tissues of FMDV-carrier cattle compared to controls, we have demonstrated a general trend of suppression of transcription of most genes examined including type I and II IFN and various interleukins. Although the effects were not statistically significant, there was an undeniable lack of immunostimulation which is often an expected result of a pathogen infection and has been previously reported for FMDV [26–29]. Further elucidation of viral modulation of host factors associated with the carrier state will reveal mechanisms that may be targeted in order to prevent or cure FMDV persistence. Such efforts are currently underway in our laboratory.
